# Corrosion Behavior of Titanium Dental Implants with Implantoplasty

**DOI:** 10.3390/ma15041563

**Published:** 2022-02-19

**Authors:** Pablo Lozano, Marta Peña, Mariano Herrero-Climent, Jose Vicente Rios-Santos, Blanca Rios-Carrasco, Aritza Brizuela, Javier Gil

**Affiliations:** 1 Department of Periodontology, Dental School, University of Seville, 41009 Seville, Spain; pablolozanoperezbarquero@gmail.com (P.L.); marta.pena@gmail.com (M.P.); jvrios@us.es (J.V.R.-S.); brios@us.es (B.R.-C.); 2 Department of Periodontology, Porto Dental Institute, 4150-518 Porto, Portugal; dr.herrero@herrerocliment.com; 3 Departamento de Cirugía y Especialidades Médico-Quirúrgicas, Universidad de Oviedo, 33006 Oviedo, Spain; aritzabrizuela@hotmail.com; 4 Bioengineering Institute of Technology, Facultad de Medicina y Ciencias de la Salud, Universitat Internacional de Catalunya, Sant Cugat del Vallés, 08195 Barcelona, Spain; 5 Faculty of Dentistry, Universitat Internacional de Catalunya, Sant Cugat del Vallés, 08195 Barcelona, Spain

**Keywords:** corrosion, titanium ion release, implantoplasty, debris, residual stresses

## Abstract

The procedure generally used to remove bacterial biofilm adhering to the surface of titanium on dental implants is implantoplasty. This treatment is based on the machining of the titanium surface to remove bacterial plaque. In this study, we used 60 grade 4 titanium implants and performed the implantoplasty protocol. Using X-ray diffraction, we determined the stresses accumulated in each of the as-received, machined and debris implants. The resistance to corrosion in open circuit and potentiodynamically in physiological medium has been determined, and the corrosion potentials and intensities have been determined. Tests have been carried out to determine ion release by ICP-MS at different immersion times. The results show that the corrosion resistance and the release of titanium ions into the medium are related to the accumulated energy or the degree of deformation. The titanium debris exhibit compressive residual stresses of −202 MPa, the implant treated with implantoplasty −120 MPa, and as-received −77 MPa, with their corrosion behavior resulting in corrosion rates of 0.501, 0.77, and 0.444 mm/year, respectively. Debris is the material with the worst corrosion resistance and the one that releases the most titanium ions to the physiological medium (15.3 ppb after 21 days vs. 7 ppb for as-received samples). Pitting has been observed on the surface of the debris released into the physiological environment. This behavior should be taken into account by clinicians for the good long-term behavior of implants with implantoplasty.

## 1. Introduction

The growth of bone tissue around dental implants has proven to be a highly successful treatment. The levels of osseointegration normally achieved are between 65% and 80% of bone in contact with the dental implant, giving a good mechanical fixation [1,2,3,4,5,6]. However, currently, the most common danger to osseointegration is peri-implantitis caused by bacterial colonization. According to the consensus meeting of Periodontology held in 2008, peri-implant mucositis can be defined as the presence of inflammation of the peri-implant mucosa without signs of loss of bone support, while peri-implantitis, in addition to inflammation of the mucosa, is characterized by a loss of bone support [7,8,9]. The main factor associated with peri-implantitis is the bacterial plaque around implants due to a lack of oral hygiene, tobacco, or previous history of periodontal disease [10,11,12,13,14].

When the biofilm adheres to the implant surface, it can produce peri-implant mucosal inflammation and if the surface is untreated, it can provoke the loss of peri-implant bone. Several authors [15,16,17] studied how peri-implantitis presents a site-specific infection and has many features in common with chronic adult periodontitis.

**Figure 1:**
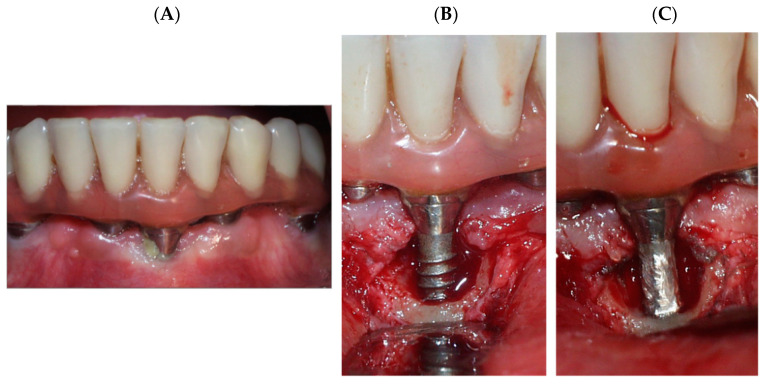
(**A**) Dental implant with peri-implantitis, (**B**) Resective surgery. (**C**) Polishing of the titanium surface to remove plaque. Implantoplasty process.

Several surgical techniques, such as regenerative, access, or respective surgeries, or a combination of these, have been proposed for the peri-implantitis treatment [15,16]. On many occasions after the surgery, the roughness surface may be in contact with the oral medium. In these cases, implantoplasty (IP) can be applied in order to decontaminate the supracrestal component of the implant. This technique consists of polishing the implant surface that has been uncovered by the loss of peri-implant bone (Figure 1) [17]. During IP, there is a large release of metal debris into the peri-implant tissue that often cannot be completely removed and remains in the bone and mucosal tissue around the implant. Besides, the implant machined presents a loss of mechanical properties and presents a new topography, which is not favorable to osteoblast adhesion. Several researchers studied that Ti debris in the peri-implant tissue could trigger peri-implant bone loss [14,15,16]. In addition, Soto-Alvaredo et al. [18] observed that Ti nanoparticles and ions exert a cytotoxic effect upon human enterocytes and murine osteoblasts [18,19,20]. However, the impact of such metal debris released during IP on a living organism remains unclear. In this contribution, the corrosion resistance and titanium ion release have been studied in an as-received dental implant, once the dental implant has been polished (machined) and the debris produced.

In this contribution, we have studied the corrosion behaviour of the original titanium implants after implantoplasty as well as the debris produced by machining. In addition to corrosion, the residual stresses on the surface of the dental implant generated by machining have been determined. These stresses have an important influence on the chemical degradation behaviour of titanium. These results should be taken into account by clinicians for the long-term performance of titanium.

## 2. Materials and Methods

**Figure 2:**
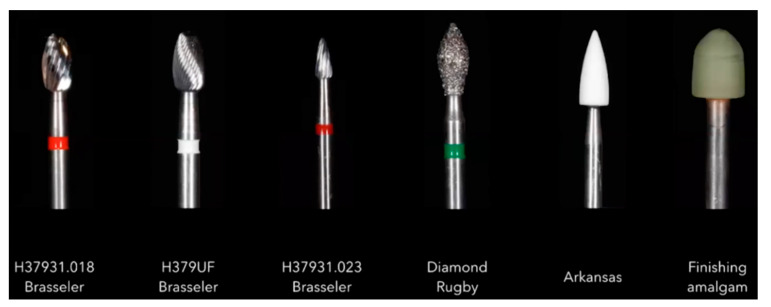
Instruments used for the implantoplasty.

Implantoplasty of 60 commercially pure Ti (grade 4) dental implants (Vega, Klockner, Escaldes Engordany, Andorra) was carried out by the same investigator (MP) using a GENTLEsilence LUX 8000B turbine (KaVo Dental GmbH, Biberach an der Riß, Germany) with water irrigation at 20 °C. The surface was sequentially polished with a small-grained WC bur (reference H37931. 018 followed H37UF and H37931023, (Brasseler, KOMET; GmbH & Co., KG, Lemgo, Germany), a coarse-grained diamond polisher (Rugbyno. 9608.314.030 KOMET; GmbH & Co., KG, Lemgo, Germany), and a small-grained SiC polisher Arkansas and finishing amalgam (order no. 9618.314.553 KOMET; GmbH & Co., KG, Lemgo, Germany). For each drill, the time used was 1 min. In Figure 2, all the instruments used in the implantoplasty process can be observed.

In order to dry the water from the metal particles, the samples were lyophilized. Once 10 g of Ti powder was obtained with original dental implant and dental implant modified by the implantoplasty procedure, the residual stress, open circuit corrosion, potentiodynamic curves, and titanium ion release were studied.

The samples studied comprised three groups:

- Cp-Ti dental implant as received. (grade 4). (As-received)
- Cp-Ti dental implant treated by implantoplasty. (Implantoplasty)
- Cp-Ti debris obtained by implantoplasty. (Debris)

Surface residual stresses were determined by an x-Ray diffractometer with a Bragg-Bentano configuration (D500, Siemens, Munchen, Germany). The stresses were calculated for the family of planes (213), which diffract at 2θ = 139.5°. With these crystallographic conditions, the elastic constants are EC = (E/1 + υ)(213) = 90.3 (1.5) GPa. Τhe angles (θ) determined at 0° and five negative- and five positive-angles. Eleven angles were evaluated. The peaks were adjusted with a pseudo-Voigt function using special software (WinplotR, free access on-line) and then transformed to interplanar distances (dψ) using Bragg’s equation. The dψ vs. sen^2^ψ graphs and the calculation of the slope of the linear regression (A) were obtained by means of adequate software (Origin, Microcal, Wellsley, MA, USA). The residual stress is: σ = EC*(1/d0)*A, where d0 is the interplanar distance for ψ = 0°.

A total of 30 samples, 10 samples per group, were used for the corrosion tests. The test area for each sample was 19.6 mm^2^. The electrolyte for all tests was Hank’s solution (ThermoFisher, Madrid, Spain) (Table 1), which is a saline fluid that closely captures the ion composition of the human serum environment [4,6].

**Figure 3:**
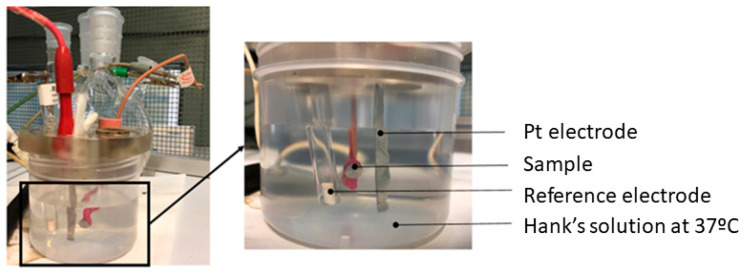
Corrosion resistance equipment.

The electrochemical cell used was a polypropylene (PP) container with a capacity of 185 mL and a methacrylate lid with 6 holes for the introduction of the sample, the reference electrode, and the counter electrode (Figure 3). The reference electrode was calomel (saturated KCl) for open circuit potential and potentiodynamic tests. The potential of this electrode is 0.241 V compared with the standard hydrogen electrode. All tests were performed at room temperature and in a Faraday box.

The calomel electrode and the sample were placed in the electrochemical cell to determine the corrosion potential results in open-circuit. Measurements were analysed for 5 h, taking results every 10 s. The potential was accepted when the variation of the potential is lower than 2 mV for 30 min in accordance with the ASTM G31 standard [20,21,22,23,24]. This test assesses which materials are more noble (higher potential), and thus less susceptible to corrode. The data and the E-t curves were obtained using the PowerSuite software with the PowerCorr-Open circuit.

Potentiodynamic polarisation curves were obtained for the 3 study groups taking in account the ASTM G5 standard [23]. In this test, a variable electrical potential is imposed by the potentiostat between the calomel electrode and the sample, producing a current to flow between the sample and the counter electrode. The counter electrode used was platinum [24]. Initially, the system was allowed to stabilise by means of an open-circuit test for 1 h. After stabilization, this test was launched, performing a cyclic sweep from −0.8 mV to 1.7 mV at a speed of 2 mV/s. These parameters were registered into the PowerSuite software and the PowerCorr-Cyclic Polarization function can be used to obtain the graphs. The results studied were:

- i_corr_ (μA/cm^2^)/corrosion current density.
- E_corr_ (mV)/Corrosion potential: value at which the current density changes from cathodic to anodic.
- E_rep_ (mV)/Repassivation potential: potential at which the passive layer regenerates.
- E_p_ (mV)/Pitting potential: value at which pitting corrosion may occur.
- i_p_ (μA/cm^2^)/passivation current density.
- i_p_ (μA/cm^2^)/repassivation current density.

The E_corr_ and i_corr_ parameters are obtained by extrapolating the Tafel slopes. The Tafel slopes are also used to obtain the Tafel coefficients: anodic (βa) and cathodic (βc). These coefficients represent the slopes of the anodic and cathodic branch, respectively. In accordance with the ASTM G102-89 standard [24], these values are then used to calculate the polarization resistance (R_p_) using the Stern-Geary expression and the corrosion rate (CR in mm/year) [24,25,26,27,28].


$$R_{P}=\frac{\beta a\;\beta c}{2.303\left(\beta a+\beta c\right)i_{\mathrm{corr}}}$$

$$\mathrm{CR}=K_{1}\;\frac{i_{\mathrm{corr}}}{\rho }\;\mathrm{EW}$$

K is the Stern-Geary constant (determined by the Tafel constants) is the only variable that is normally not measured, but commonly assumed to be a value of 0.025. EW is the equivalent weight. For an atomic species (so pure metals), the equivalent weight EW is the atomic weight AW divided by the number of electrons needed for conversion. The polarisation resistance indicates the resistance of the sample to corrosion when subjected to small variations in potential. A total of 30 potentiodynamic tests were carried out, obtaining at least 10 curves per group.

Five samples from each group have been used for the metal ion recovery test. After weighing the samples (m = 0.206 g) and following the ISO 10993-12 standard [23], a weight adjustment was made at the rate of 1 mL of Hank’s solution for each 0.20 g of sample, as indicated in the standard. The 5 samples of each group were placed in the same Eppendorf with 5 mL of Hank’s solution and stored at 37 °C. Hank’s solution should be extracted and stored in the refrigerator after 1, 3, 7, 14, and 21 days. After each extraction, 5 mL of fresh Hank’s solution was replenished in the Eppendorf containing the samples. All Eppendorf tubes should be cleaned with 2% nitric acid and dried before use.

After 21 days, the concentration of released titanium ions was measured, at the test times indicated above, by inductively coupled plasma mass spectrometry (ICP-MS) with the Agilent Technologies 7800 ICP-MS.

## 3. Results

**Figure 4:**
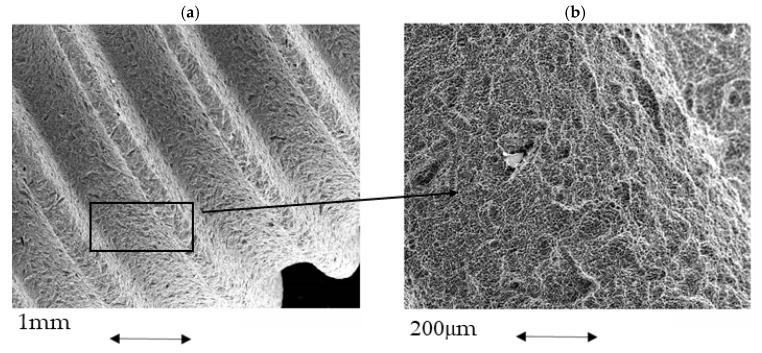
(**a**) As-received titanium dental implant. (**b**) Passivated surface on the titanium.

Figure 4 shows the rough surface of the original dental implant by electron microcopy. The roughness determined was Ra of 1.87 μm, which is the most suitable roughness for osseointegration [2,3,4]. This roughness was obtained by shot blasting using Al_2_O_3_ as the abrasive element.

**Figure 5:**
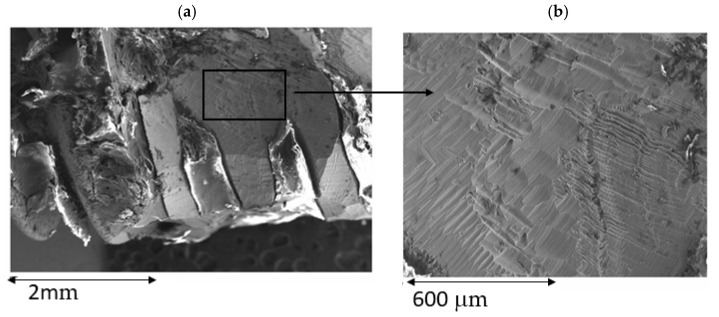
(**a**) Titanium dental implant after implatoplasty porcess. (**b**) Slippimg bands produce by the high stress applied.

**Figure 6:**
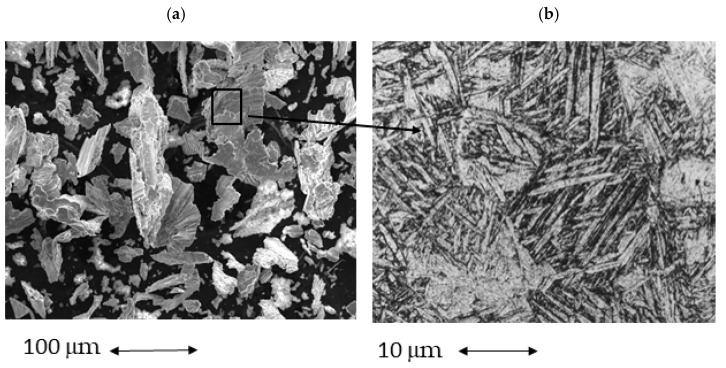
(**a**) Titanium debris produced by implantoplasty in titanium dental implant. (**b**) Widmanstatten microstucture of cp Ti with high deformation.

Figure 5 shows a dental implant that has been treated by implantoplasty, where we can observe the great damage that is exerted on the dental implant. In this case, the rough area disappears due to machining, leaving a smoother surface but full of machining scratches. In Figure 5, at greater magnification, one can see the sliding lines or deformation bands, indicating the large amount of deformation on the surface. In Figure 6, the particles are detached from the dental implant and can be are observed in the form of an arc due to the deformation produced, which in this case has exceeded the fracture stress. Figure 6 at greater magnification shows the microstructure of equiaxial titanium grains with large deformation bands as well as twinning on the inside of the crystals, which shows the high internal stress of these particles. This microstructure has been revealed by HF treatment for 15 s.

The values for the residual stresses are summarized in Table 2. Compressive stresses induced by implantoplasty on c.p. Ti are statistically significant (*p* < 0.001, t-Student) and highly different from as-received dental implant with the debris. All the results present statistically significant differences between as-received, implantoplasty, and debris.

**Figure 7:**
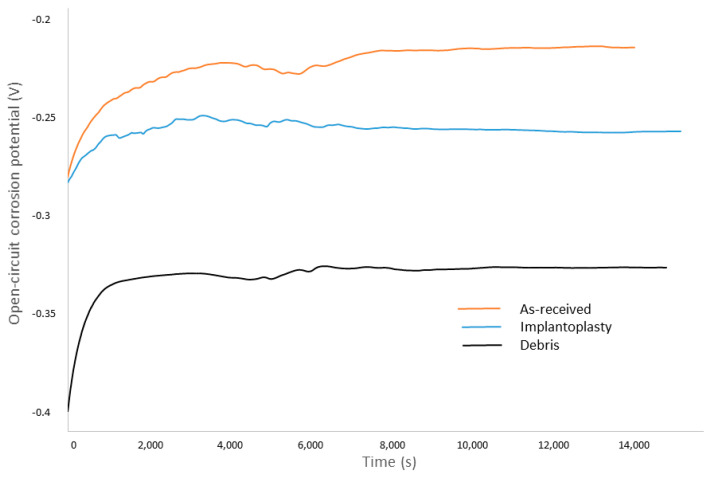
Open-circuit corrosion potential versus time of immersion.

For the corrosion studies, the results can be observed in Table 3. These results show that the highest open-circuit corrosion potential values (E_OCP_) were obtained for as-received dental implant. Conversely, the titanium debris showed the lowest values in open circuit, which indicated the highest tendency for corrosion. In Figure 7, the different curves of open-circuit corrosion potential in relation to the time can be observed.

**Figure 8:**
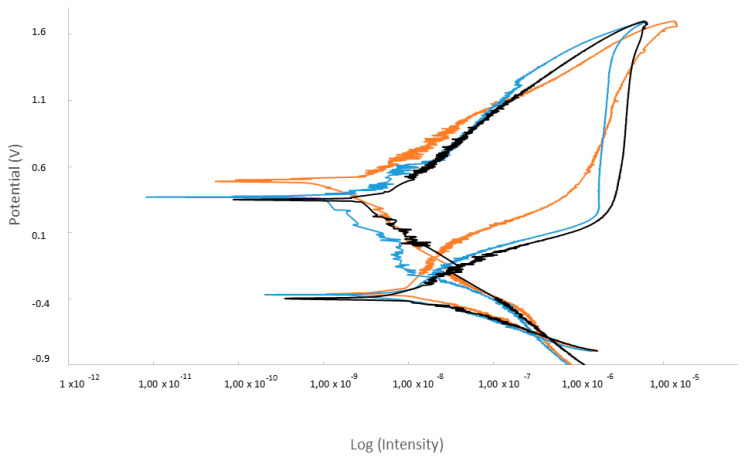
Potentiodynamic curves for the different samples studied.

The potentiodynamic analysis confirmed that the treatment that produced surfaces with the best corrosion resistance was as-received dental implants, showing the lowest values of corrosion current density (i_corr_) and corrosion rate (V_c_). The curves are shown in Figure 8. In addition, the original implants show the highest resistance to polarization (R_p_). Implantoplasty produced a loss of the corrosion resistance with respect to the as-received samples.

**Figure 9:**
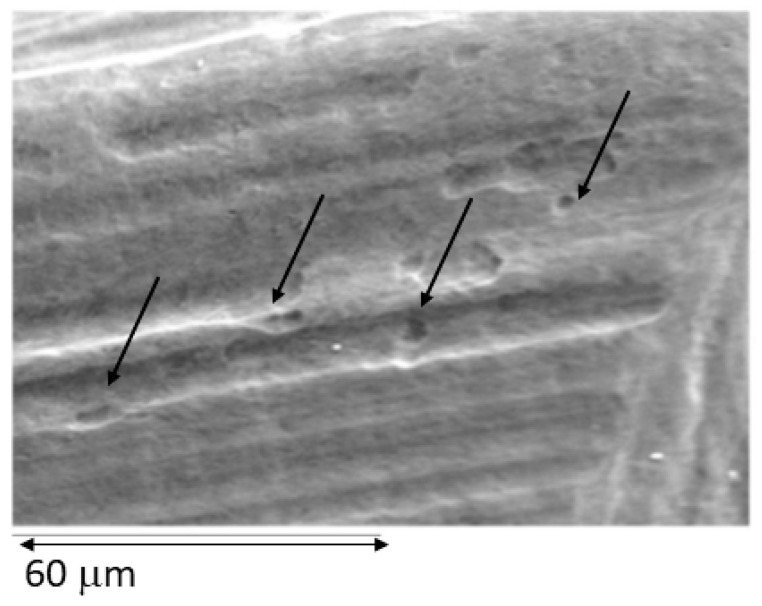
Pitting corrosion marks produced after completing the potentiodynamic test on a debris titanium released aby the implantoplasty process.

Moreover, only in titanium debris has pitting been observed by scanning electron microscopy. This fact is due to the aggressive corrosion behavior and favoured the degradation of titanium in two ways, namely titanium ion release and the oxidation of the particles. In Figure 9, the pitting of the debris can be observed.

Table 4 shows the cumulative Ti ion release in parts per billion (ppb) from the different samples studied in Hank’s solution after increasing days of incubation. Analogous to the highest electrochemical stability, Ti ion release was the lowest from as-received, with a total cumulative concentration after 21 days of incubation of 7.0 ± 0.6 ppb. Differences are statistically significant when comparing Ti ion release from as-received surfaces with titanium debris (15.3 ppb ± 1.9). More than doubled the ion release values from as-received surfaces.

## 4. Discussion

One of the main problems in oral implant dentistry is peri-implantitis and it is currently the leading cause of revision. The absence of bactericidal treatments approved by accreditation agencies due to the long-term nature of clinical studies of different bactericidal implant strategies [29,30,31,32,33,34,35] has given rise to treatments such as implantoplasty that can be performed in any dental clinic. Bactericidal treatments under study include silver nanoparticles as an element with a high bactericidal capacity [30,31], the functionalisation of titanium with organic compounds, such as TESPSA and lactoferrin among others, and the use of titanium with organic compounds, such as TESPSA and lactoferrin [31,36]. The long-term behavior of nanoparticles or the complicated and costly anchoring of organic compounds in titanium means that these treatments have not yet matured sufficiently for their application. This has led to implantoplasty being applied at least in the most severe cases. However, there are no protocols and there are not many studies of the implications of such an aggressive treatment as implantoplasty.

Undoubtedly, the machining of titanium causes a severe loss of mechanical properties due to the reduction of the area of the dental implant and the important plastic deformation produced in the titanium. In addition, the effect of machining on the anchorage points to the host tissue. In this aspect, implantoplasty should be discouraged in narrow dental implants as the sectioning in these is very compromised and there could be a danger of fracture of the dental implant [37,38].

In addition to the mechanical damage, there is also damage to chemical stability. In this contribution, we have been able to verify the loss of corrosion resistance of the machined implant and even more so in titanium residues. As we have seen, the loss of corrosion resistance goes hand in hand with an increase in the mechanical stress accumulated in the material. It is well known that metals with high stored energy are very susceptible to corrosion. In this study, it was found that the debris, which has the highest stored energy due to the large amount of deformation, is the most corrosive. This fact is worrying as these particles can remain in the surrounding tissues and also produce titanium oxide. It is well known that there are many different stoichiometries of titanium oxide that are not biocompatible [39,40]. In addition, if the particles are very small in the nanometer size range, they are not detectable by the immune system and can lead to more serious systemic problems [40,41,42,43]. There is already some work on the reaction of inflammatory cells to the detached particles and the results are not very good. Therefore, implantoplasty should be considered as a risky treatment and should be performed in cases of severe bacterial colonization with bone loss. Researchers in the field of biomaterials, together with clinicians, must work to achieve a bactericidal treatment that avoids such an aggressive treatment as implantoplasty.

Recently, Toledano-Serrabona et al. [44,45] have studied the characteristics of detached particles in Ti-6Al-4V dental implants showing a certain level of cytotoxicity and inflammatory cell reaction. In addition, the surface properties of the dental implant topography have been studied [46] and the difficulty of osteoblastic growth on the surfaces of the dental implant with implantoplasty. Likewise, strategies have been studied to prevent infection processes in implantoplasty treatments with the introduction of antibiotics [47]. The combination of a resective and reconstructive surgical approach together with locally delivered antibiotic achieved a high disease resolution rate after one year of follow-up and constitutes a viable option for the management of peri-implantitis a short-term. The results are encouraging, but they do not have a long-lasting bactericidal action. Antibiotics also have no effect on the toxicity processes of the particles and the surface of the dental implant in the case of corrosion, as antibiotics have no effect.

Indeed, the technique of implantoplasty removes biofilms from the titanium surface to prevent peri-implantitis and also preserves the osseointegrated dental implant. However, as we have seen, it reduces the resistance to corrosion, increases the release of titanium ions into the medium, and reducing the implant cross-section by machining will lead to a reduction in mechanical properties. In this sense, it is necessary to continue researching new anodisation treatment techniques, such as those published by Harraft et al. [48] where titanium is anodised in the form of TiO_2_ nanotubes that can contain bactericidal drugs, or others based on citric acid [49], or with Pirahna treatments that, due to their nanotexture, prevent bacterial colonization [50].

One aspect that should be studied is how the titanium surface will behave towards bacteria after implantoplasty. In principle, everything suggests that having a surface with less roughness than that of the as-received implant will make it more difficult for biofilm to form [30,31]. It is well known that the roughness of the dental implant, which is fundamental for osseointegration and fixation of the implant to the bone, is detrimental to bacterial colonization [4,5]. Therefore, the reduced roughness caused by the machining of the implant process would favour the reduction of plaque adhesion. In the same way, the lack of a certain roughness should be studied if osteoblasts could adhere, proliferate, and differentiate on this surface. It will undoubtedly be a disadvantage for the formation of new bone. For all these reasons, it is necessary to continue studying these biological aspects on titanium surfaces that have undergone implantoplasty.

## 5. Conclusions

The implantoplasty technique is used to remove bacterial plaque adhering to the titanium surface. The machining of the dental implant causes a high residual stress on the surface of the dental implant, which promotes electrochemical corrosion. In addition, the particles released into the medium have higher stored energy and therefore greater susceptibility towards corrosion. For debris, the highest values of corrosion rate and release of ions into the physiological medium are observed. Ion release behaves analogously to corrosion resistance. Pitting has been observed in the debris. This behavior is of clinical relevance and we must be careful with the techniques of particle removal in the oral environment.

## Figures and Tables

**Table 1:** Chemical composition of Hank’s solution.

Chemical Product	Composition (mM)
K_2_HPO_4_	0.44
KCl	5.4
CaCl_2_	1.3
Na_2_HPO_4_	0.25
NaCl	137
NaHCO_3_	4.2
MgSO_4_	1.0
C_6_H_12_O_6_	5.5

**Table 2:** Surface residual stresses calculated at the different samples.

Material	σ (MPa)
As-received	−77.2 ± 5.2
Implantoplasty	−120.0 ± 10.3
Debris	−202.1 ± 12.2

**Table 3:** Electrochemical and corrosion parameters assessed for Ti alloy meshes with different passivation treatments.

Samples	E_OCP_ (mV)	i_corr_ (μA/cm^2^)	R_p_ (MΩ/cm^2^)	E_CORR_ (V)	V_c_ (mm/year)
As-received	−195 ± 9	0.049 ± 0.007	1.14±0.11	−340 ± 32	0.444±0.067
Implantoplasty	−273 ± 10	0.056 ± 0.005	1.07±0.18	−368 ± 47	0.477±0.045
Debris	−334 ± 17	0.063 ± 0.009	1.00±0.06	−411 ± 21	0.501±0.077

**Table 4:** Ti ion release (ppb) at different incubation times in Hank’s solution.

Mesh	1 Day	3 Days	7 Days	14 Days	21 Days
As-receieved	1.3 ± 0.2	2.7 ± 0.5	2,9 ± 0.5	4.5 ± 0.4	7.0 ± 0.6
Implantoplasty	1.7 ± 0.4	3.3 ± 0.4	4.1 ± 0.2	5.7 ± 0.3	9.1 ± 0.5
Debris	2.9 ± 0.8	4.8 ± 0.8	5.3 ± 0.9	10.4 ± 5.9	15.3 ± 1.9

## Data Availability

Not applicable.
